# Novel Analysis of Oceanic Surface Water Metagenomes Suggests Importance of Polyphosphate Metabolism in Oligotrophic Environments

**DOI:** 10.1371/journal.pone.0016499

**Published:** 2011-01-28

**Authors:** Ben Temperton, Jack A. Gilbert, John P. Quinn, John W. McGrath

**Affiliations:** 1 School of Biological Sciences, Queen's University Belfast, Belfast, United Kingdom; 2 Institute of Genomic and Systems Biology, Argonne National Laboratory, Argonne, Illinois, United States of America; 3 Department of Ecology and Evolution, University of Chicago, Chicago, Illinois, United States of America; 4 Plymouth Marine Laboratory, Plymouth, United Kingdom; Miami University, United States of America

## Abstract

Polyphosphate is a ubiquitous linear homopolymer of phosphate residues linked by high-energy bonds similar to those found in ATP. It has been associated with many processes including pathogenicity, DNA uptake and multiple stress responses across all domains. Bacteria have also been shown to use polyphosphate as a way to store phosphate when transferred from phosphate-limited to phosphate-rich media – a process exploited in wastewater treatment and other environmental contaminant remediation. Despite this, there has, to date, been little research into the role of polyphosphate in the survival of marine bacterioplankton in oligotrophic environments. The three main proteins involved in polyphosphate metabolism, Ppk1, Ppk2 and Ppx are multi-domain and have differential inter-domain and inter-gene conservation, making unbiased analysis of relative abundance in metagenomic datasets difficult. This paper describes the development of a novel Isofunctional Homolog Annotation Tool (IHAT) to detect homologs of genes with a broad range of conservation without bias of traditional expect-value cutoffs. IHAT analysis of the Global Ocean Sampling (GOS) dataset revealed that genes associated with polyphosphate metabolism are more abundant in environments where available phosphate is limited, suggesting an important role for polyphosphate metabolism in marine oligotrophs.

## Introduction

Phosphate is a critical nutrient for the growth and function of cellular life, used in creation of phospholipids, nucleic acids and powering metabolism via adenosine triphosphate (ATP). The importance of phosphate in all metabolic pathways is such that it is believed to be the ultimate limiting nutrient for marine primary productivity in surface waters [1], [2]. Recently, there has been significant focus on the potential of marine bacterioplankton to utilize dissolved organic phosphates (DOP), present in concentrations dwarfing those of inorganic phosphates [3]. It is believed that DOP utilization could alleviate phosphate-limitation via alkaline phosphatase cleavage of phosphoesters (C-O-P bonds) [4]–[7] and via cleavage of phosphonates (C-P bonds) [8]–[11]. To date, however, there has been little research into the role of polyphosphate metabolism in marine bacterioplankton. Polyphosphate is a linear homopolymer of phosphate residues linked together by high-energy phosphoanhydride bonds, similar to those found in adenosine triphosphate (ATP). It is one of the most widely distributed macromolecules and has been found in all forms of life in all three domains [12], accounting for up to 10−20% of the dry weight of a cell in certain bacteria [13]. Synthesis proceeds via the polymerization of the terminal phosphate of ATP, catalyzed by polyphosphate kinase (Ppk1)[12]. Polyphosphate has been identified as important in a number of metabolic pathways including P_i_ storage under nutrient limitation, controlling viral burst size, horizontal gene transfer and regulation of pathogenicity [14]. Furthermore, its potential for biotechnological exploitation as an ATP substitute [15] and in remediation of environmental contaminants [16]–[18] highlight it as a good candidate for bioengineering.

Under nutrient limitation, polyphosphate metabolism serves three main functions in bacteria. Firstly, polyphosphate acts as a regulator of the stringent response by controlling levels of sigma factors and through binding to RNA polymerase, inhibiting transcription of genes involved in exponential growth [19]. Upon return to a nutrient rich environment, polyphosphate is degraded, re-enabling expression. Polyphosphate can also act as a storage mechanism for phosphate via a mechanism known as ‘polyphosphate overplus’ [20]. When shifted from phosphate-limited to phosphate-rich media certain bacterial strains increase their internal polyphosphate concentrations by as much as 150-fold [21]. Given that polyphosphate can act as a direct substitute for ATP for many kinases [12], the overplus mechanism can massively increase the energy stores of the cell. In oligotrophic environments, where nutrient availability is sporadic, the ability to rapidly accumulate polyphosphate for survival of P_i_-limitation would be favorable. Accumulated polyphosphate can either be degraded to ATP via the reverse reaction of Ppk1, or released as inorganic phosphate (P_i_) via an exopolyphosphatase (Ppx) encoded by a gene that often neighbors *ppk1* in an operon [22], [23]. Polyphosphate may also be degraded to generate GTP, important in the stringent response, via the phosphorylation of GDP, catalyzed by polyphosphate kinase 2 (Ppk2). It is also possible that accumulation of P_i_ as polyphosphate may postpone cellular saturation of free P_i_ by acting as a P_i_ sink. This would kinetically drive the uptake of P_i_ by the high-affinity transporter PstS, which has been shown to dominate the metaproteome in P_i_-limited systems [24]. A secondary benefit of such a system would be the osmotically neutral storage of cations, associated with the electron-dense polyphosphate polymer. Consumption of the polyphosphate chain would release protonated metal chelates, generating membrane potential that could be utilized in both ATP synthesis [25] and substrate uptake [26]. The aim of this research was to examine the prevalence of polyphosphate metabolism genes in the metagenomic samples taken as part of the Global Ocean Sampling Expedition.

**Figure 1:**
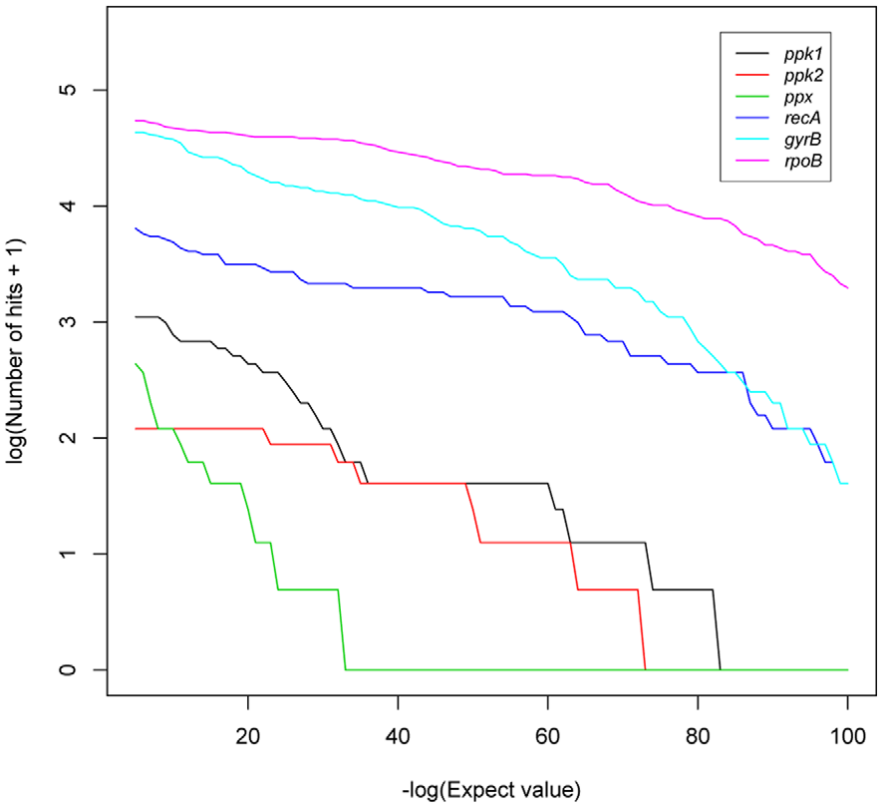
Effect of different expect-value cutoffs on the number of identified homologs in a subsampled nucleotide database from the GOS expedition, using amino acid sequences from *Pseudomonas aeruginosa* PAO1 as a query in a TBLASTN local alignment search. *ppk1*, *ppk2* and *ppx* are polyphosphate metabolism genes comprising of regions of both high and low conservation. *recA*, *gyrB*, and *rpoB* are single copy marker genes and tend to be highly conserved.

The Global Ocean Sampling Expedition (GOS) represents the largest marine metagenomic dataset to date, comprising 6.3 billion basepairs of genomic information across 44 sites in its first phase alone [27]. Consequently, it has been pivotal in attempts to improve understanding of microbial functional metabolism through bioinformatic analysis [4], [6], [10], [27]–[29]. Such studies have often used local alignment algorithms such as BLAST [30] to identify gene fragments sharing isofunctional homology to a known reference sequence, paired with selective expect-value cutoffs and/or reciprocal best-hit protocols to exclude hits deemed too dissimilar to have a statistical likelihood of homology. However, this approach is not without pitfalls. When comparing relative abundance of one gene to another, either a secondary functional gene [4], or a single-copy marker gene for estimations of per-cell abundance [6], it cannot be assumed that the same expect-value cutoff will equally affect the inclusion or exclusion of putative homologs for different genes. Effect of expect-value cutoff is a function of gene conservation, conserved domain number and gene length. This is demonstrated in **Figure 1**, which shows that the number of single-copy marker gene homologs (*recA*, *gyrB* and *rpoB*) identified by BLAST when compared against a re-sampled GOS dataset decreases more slowly with a decreasing expect-value cutoff compared with less well-conserved genes. Consequently, the choice of expect-value cutoff will affect any downstream analysis of relative gene abundance. To circumvent this bias, a more inclusive expect-value is often combined with a filtering of putative homologs by crosschecking against a second protein database, known as a reciprocal-BLAST. The National Center for Biotechnology Information (NCBI) non-redundant CDS translation database is often used for this task. A putative homolog from the first search is classed as a homolog if one of the closest matches in the second search matches the annotation of the query sequence [31], [32]. However, as environmental genomic studies continue to increase exponentially the number of sequences from either putatively annotated or hypothetical proteins from uncultured bacteria in such databases will also increase dramatically. This makes it more difficult to successfully crosscheck hits using a reciprocal BLAST as the number of unknown matches returned as top hits increases. This is a particular problem for multi-domain proteins where a genomic fragment may cross two domains and thus be rejected in the crosscheck if part of the fragment has a better match to a shared ancestral conserved domain on a non-homologous protein.

Multi-domain proteins can also be problematic when using Position-Specific Score Matrices (PSSM) in probabilistic inference analysis as used in HMMER3 [33]. In traditional BLAST analysis of two fragments, a non-matching sequence from a poorly conserved region is penalized as heavily as a non-matching sequence from a highly conserved region. Both HMMER3 and PSI-BLAST [34] overcome this weakness by weighting mismatch penalties on the likelihood of a match for a given region (i.e. how conserved a particular region of the gene is). These methods are often used in conjunction with a Hidden Markov Model (HMM) for a particular conserved domain, available in the PFAM database [35]. However, when searching for homologs of a multi-domain protein where one of the domains is highly ubiquitous (e.g. an ATP-binding cassette), a large number of true homologs (in the sense that the domains share a common ancestor) will be returned which are not necessarily isofunctional (the terms ‘ortholog’ and ‘paralog’ have been deliberately avoided in this manuscript due to the potential confusion arising from these terms as highlighted by Jensen [36]). Thus, to identify isofunctionally homologous sequences, a reciprocal BLAST step would be required to filter out any hits that shared one or more domains with the query but which also contained non-matching domains, suggesting a different function.

Given that the three genes involved in polyphosphate metabolism, *ppk1*, *ppk2* and *ppx* are all multi-domain and contain ubiquitous ATP binding sites coupled with regions of variable conservation [37], [38], it was felt that a new pipeline was required to successfully annotate isofunctional homologs of these genes from a metagenomic dataset. The requirements of the pipeline were four-fold: (i) The pipeline had to be free from bias resulting from arbitrary expect-value cutoffs, as *ppx* is more poorly conserved than *ppk1* and *ppk2* (**Figure 1**). (ii) The pipeline was required to consistently annotate isofunctional homologs of these genes across multiple marine metagenomic datasets, using unassembled reads from both Sanger (∼1000 basepair (bp)) and GS-FLX (∼350 bp), so that their abundances could be accurately compared. (iii) It had to implement a PSSM-based homology search using PSI-BLAST. (iv) Annotated putative homologs required a reciprocal homology check against a well-curated database free from hypothetical proteins of uncultured species. This paper describes a novel Isofunctional Homolog Annotation Tool (IHAT) developed to meet these criteria. In an evaluation against other homology search methods, IHAT performed better at annotating isofunctional homologs and avoiding heterofunctional homologs than BLAST or HMMER3 and did not require prior translation of nucleotide sequences into putative Open Reading Frames (ORFs).

Analysis of the GOS dataset using IHAT revealed a strong correlation between *ppk1* and *ppx* abundance and estimated phosphate concentrations. *ppk2* abundance did not correlate with P_i_ concentration, nitrate/nitrite concentration, temperature or salinity. These results suggest that polyphosphate synthesis and degradation may confer a selective advantage to oligotrophs in P_i_-limited environments. Furthermore, the genes involved in organic and inorganic phosphate sensing, uptake and storage used in this study serve as a model for other complex regulatory systems, suggesting that IHAT has broad applicability to other metabolic systems.

## Results

### IHAT analysis

**Figure 2:**
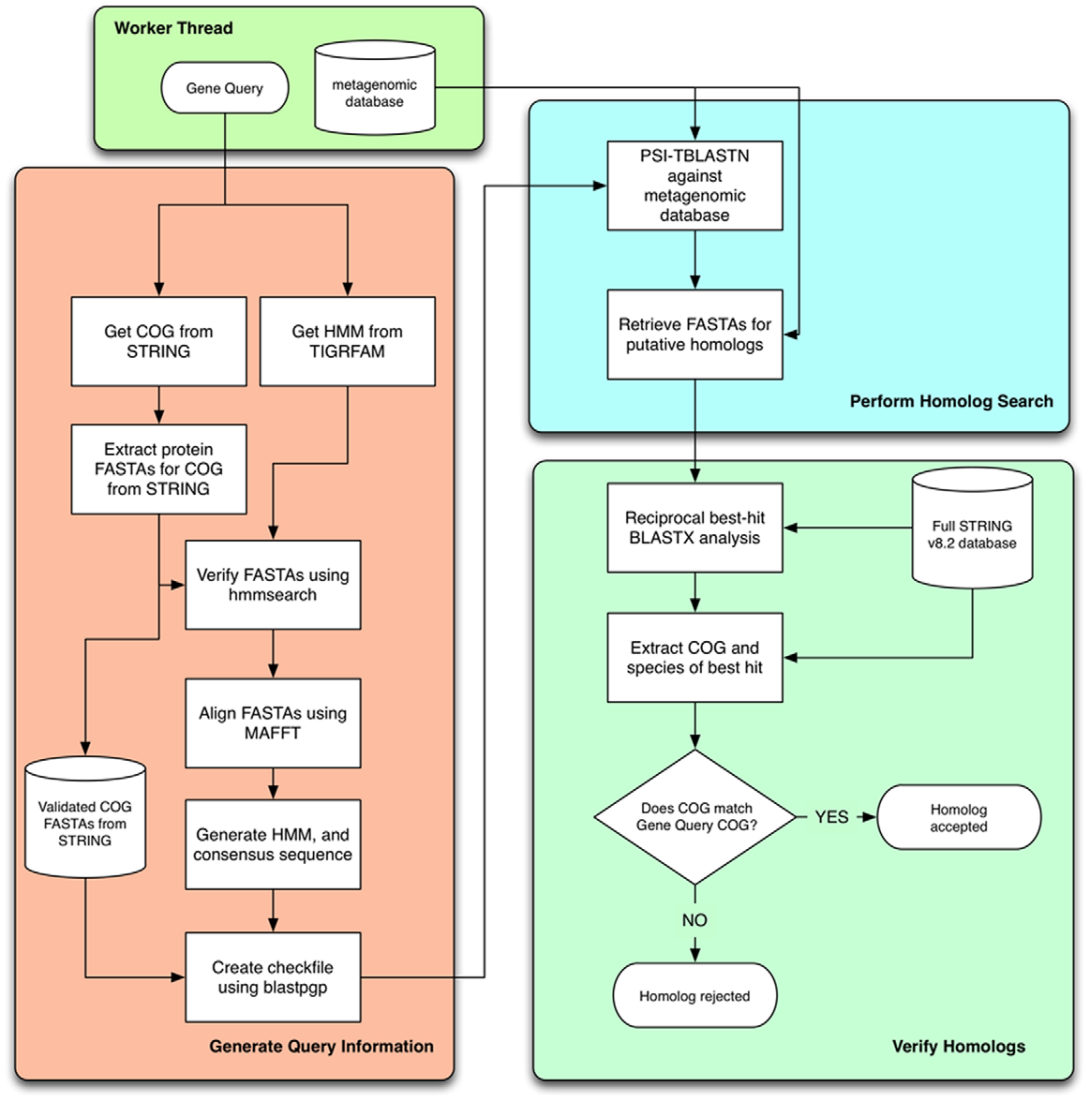
Diagrammatic representation of homolog annotation in IHAT. A worker thread is created for each combination of gene/database to be analyzed. The three colored functional blocks represent each individual stage of the process. For each gene, generation of query information only occurs once. Output from this block is reused for all subsequent homolog searches.

IHAT performs a PSI-BLAST search using a checkfile derived from a custom HMM and consensus sequence created from all members associated with a particular Clustered Orthologous Group (COG) [39] within the STRING database [40] (**Figure 2**). The STRING database was chosen as it contains detailed protein-protein interactions, enabling the identification and removal of fused genes from the model alignment to improve HMM quality. Putative homologs from this stage were verified with a reciprocal BLAST against the STRING database to check that their best-hit was also a member of the same COG. Prior to analysis of the GOS and WEC datasets, the efficacy of IHAT was tested against homolog identification using TBLASTN and HMMER3 for 11 genes chosen to cover a broad range of conservation and lengths, with known isofunctional and heterofunctional homology. For these tests, artificial datasets were created using MetaSim [41] by random sampling of the genome of *Candidatus*. “Pelagibacter ubique” HTCC7211 into 1000 bp and 350 bp fragments at 20-fold coverage.

**Figure 3:**
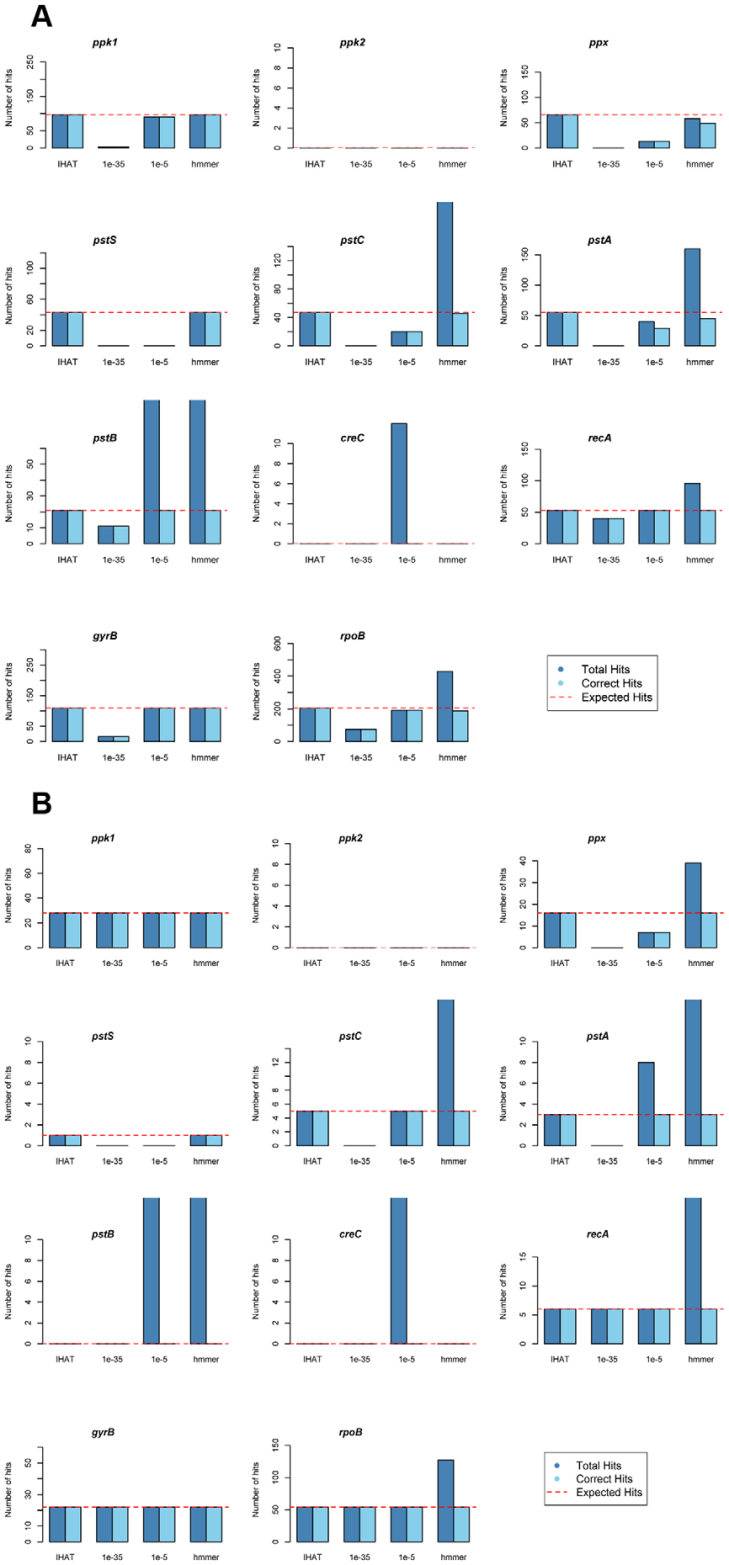
Bar plots of annotation success using the pipeline, TBLASTN with *Ps. aeruginosa* PAO1 genes at 10^−35^ and 10^−5^ expect cutoff, and HMMER3 against an artificial dataset created from HTCC7211 random (A) 350 bp and (B) 1000 bp fragments. For HMMER3 analysis, the artificial dataset was translated into ORFs using orf_finder and then scanned using HMMER3 using the STRING-generated HMMs as the query. Light blue bars represent the number of hits that were correctly annotated. Dark blue bars represent the number of correctly annotated hits plus the number of hits to other genes. The red dotted line indicates the total number of correct hits in the dataset, equal to the number of fragments sampled within a gene locus.

The comparative number of homologs returned from the 1000 bp and 350 bp *Can.* “P. ubique” HTCC7211 artificial datasets for each of the genes listed in **Table 1** is shown in **Figure 3**. The red dotted line represents the total number of fragments sampled within the start and end locus of the gene on the *Can.* “P. ubique” HTCC7211 genome. IHAT successfully identified all fragments from each gene, with no false positives in the 1000 bp or 350 bp datasets, displaying a greater sensitivity to homologs than an expect-value cutoff of 10^−35^, used in previous similar studies [4], and similar sensitivity as a cutoff of 10^−5^. TBLASTN analysis using a cutoff of 10^−5^ resulted in a high number of false positives identified for *pstA*, *pstB*, and *creC*. The lack of known homologs to *ppk2* and *creC* in the *Can.* “P. ubique” HTCC7211 genome was correctly identified by IHAT and HMMER3, with TBLASTN identifying 31 *creC* homologs at 10^−5^. Reciprocal BLASTX of these false positives against the non-redundant protein database at NCBI had a best-hit of the *chvG* gene on *Can.* “P. ubique” HTCC1062, a pH-regulated two-component sensor histidine kinase [42]. Similarly, HMMER3 analysis successfully identified expected homologs for all 11 genes, but also a large number of false positives in 6 out of 11 genes in both 1000 bp and 350 bp datasets. HMMER3 identified 918 *pstB* homologs in the 1000 bp dataset, of which 853 (∼93%) matched the top three conserved domains: PF00005 (an ABC-transporter conserved domain), PF02463 (a superfamily with ATP binding domains at the N and C termini) and PF03193 (a family of unknown function that is a member of P-loop containing NTP hydrolases) in a reciprocal hmmsearch using the PFAM-A HMM as a query. Given the length of *pstB* is 755 bp (**Table 1**), it is not possible to have 1000 bp fragments which fall between the start and end loci of this gene. 203 of the 214 homologs (∼95%) identified by HMMER3 as putative *pstC* in the 1000 bp dataset matched PF00528, a conserved region between two transmembrane domains in periplasmic substrate binding proteins. TBLASTN analysis on highly conserved genes returned the expected number of homologs with no false positives at all tested expect-value cutoffs. Surprisingly, 51 of the *recA* homologs and 73 of the *rpoB* homologs identified by HMMER3 in the 1000 bp dataset were fragments sampled from outside the locus of these genes. 49 of these *recA* homologs matched PF05729, an NTPase domain, while 68 of the 73 *rpoB* homologs matched PF04998, domain 5 of an RNA polymerase subunit encoded by *rpoC* and/or PF00623, domain 2 of a β' subunit of RNA polymerase [43] in a reciprocal hmmsearch using the PFAM-A HMM as a query.

**Table 1:** Genes used to test the efficacy of IHAT in comparison to TBLASTN alignment analysis and HMMER probabilistic inference analysis.

Gene Name	Locus tag	Product Name	Gene Length (bp)
*ppk1*	PB7211_113	Polyphosphate kinase 1	2159
*ppk2*	-	Polyphosphate kinase 2	-*
*ppx*	PB7211_261	Exopolyphosphatase	1508
*pstS*	PB7211_1190	Phosphate ABC transporter, periplasmic phosphate-binding protein	1034
*pstC*	PB7211_733	ABC transporter	1385
*pstA*	PB7211_586	Phosphate ABC transporter, permease protein	1271
*pstB*	PB7211_412	Phosphate ABC transporter, ATP-binding protein	755
*creC*	-	Sensor kinase	-*
*recA*	PB7211_1119	DNA Repair protein	1148
*gyrB*	PB7211_801	DNA gyrase, B subunit	2060
*rpoB*	PB7211_738	DNA-directed RNA polymerase, beta subunit	4085

Gene length refers to the nucleotide length of these genes in the *Can.* “P. ubique” HTCC7211 genome.

*ppk2 and *creC* have no known homologs in the *Can.* “P. ubique” HTCC7211 genome.

### Polyphosphate Gene Abundance in the GOS dataset

**Figure 4:**
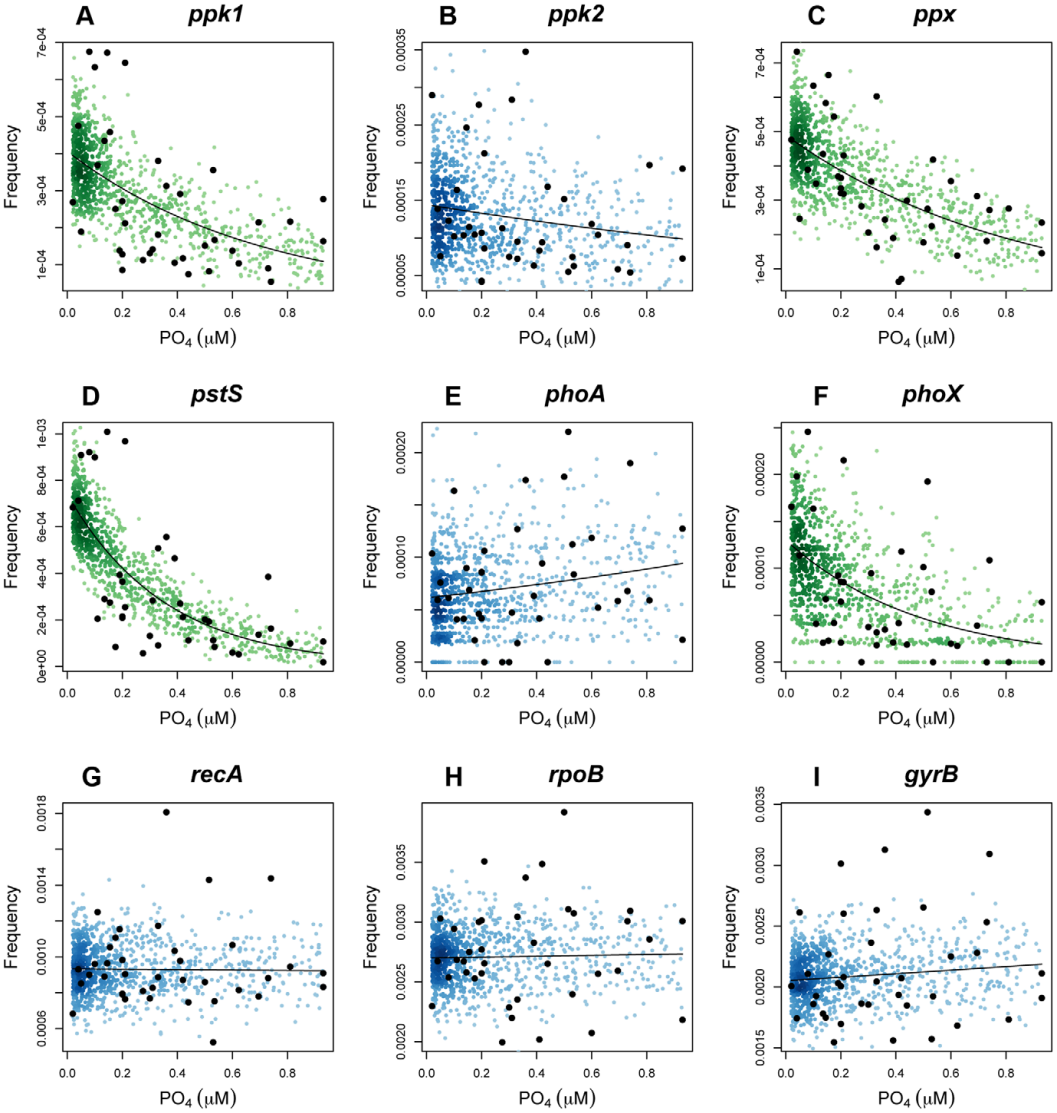
Generalized linear models of polyphosphate metabolism (A–C), high-affinity phosphate uptake (D), alkaline phosphatases (E–F) and single-copy marker gene (G–I) abundance as a function of estimated P_i_ concentration in GOS samples. Frequencies (black dots) are calculated as the number of isofunctional homologs per sequence, re-scaled to Effective Sequence Counts. Models (black lines) were created using a quasipoisson distribution. Colored dots represent a simulation of 1000 samples with equal variance and distribution to measured samples, shaded according to the local density at each point. Green dots a significant difference (*p*-value <0.05, deviance *F*-test) between the model and the null model.

IHAT was used to identify isofunctional homologs of three polyphosphate metabolism genes (*ppk1*, *ppk2*, *ppx*), the high-affinity phosphate transporter, *pstS*, the alkaline phosphatase genes *phoA* and *phoX*, and the 35 single-copy marker genes used by Raes and colleagues for calculation of effective genome size [44], across 38 GOS samples [27]. Median annual phosphate values for each GOS site were estimated using the World Ocean Database resource (see Materials and Methods). GOS samples were included in the analysis if (a) estimation of median phosphate values from the World Ocean Database could be derived from a minimum of three samples; (b) the metagenomic dataset consisted of at least 46,052 sequences; (c) the dataset was derived from the prokaryotic fraction of the filtration (0.22–0.8 µm). Sample GS033, from a hypersaline lagoon, and sample GS020, from a freshwater lake, were removed from analysis, as was sample GS025 (reef sample, different filter sizes). These samples had been previously shown to be outliers in a similarity comparison of GOS Metagenomes [45]. Samples from the initial GOS pilot study (GS000a-d) were also excluded due to an unusually high abundance of fragments identified as originating from *Burkholderia sp.* [46]. The abundance of a gene in a particular dataset was converted into the frequency an isofunctional homolog was found per sequence via Effective Sequence Count (ESC) normalization [47]. This was to remove bias from differential average genome sizes between sampled communities in GOS samples. Sampling frequencies for each gene were then modeled as a function of estimated phosphate concentration using the glm function in R [48]. Homolog abundance data was overdispersed, with dispersions ranging from 1.96 for *ppk2* to 6.55 for *pstS*. Therefore, use of a quasipoisson model was appropriate [49]. The results of this analysis can be seen in **Figure 4**. The frequency of sampling isofunctional homologs of *ppk1*, *ppx* and *pstS* showed a significant difference from the null model (*p*-value = 0.001, 9.674×10^−5^ and 3.08×10^−6^ respectively (d.f. = 37), calculated using a Fisher test analysis of deviance as is appropriate for models based on quasi-likelihood [50]). There was a clear relationship with average annual P_i_ concentrations in the GOS data. As expected, 33 of the 35 single-copy marker genes showed no correlation with P_i_ concentration, with the exceptions being *hisS* and *leuS* (*p-*values of 0.02 and 0.03 respectively, deviance *F*-test, d.f. = 37). *ppk2* showed no significant correlation between sampling frequency and P_i_ concentration (*p*-value  = 0.290, deviance *F*-test, d.f. = 37), N concentration (*p*-value = 0.830, deviance *F*-test, d.f. = 32), temperature (*p*-value = 0.431, deviance *F*-test, d.f. = 37), salinity (*p*-value = 0.896, deviance *F*-test, d.f. = 37), silicate concentration (*p*-value = 0.896, deviance *F-*test, d.f. = 37) or a model combining all five variables and their interactions (*p*-value = 0.316, deviance *F*-test, d.f. = 32). Of the two alkaline phosphatase genes, *phoX* had a significant relationship with P_i_ concentration (*p*-value = 0.003, deviance *F*-test, d.f. = 37), but *phoA* did not (*p-*value = 0.313, deviance *F-*test, d.f. = 37).

**Figure 5:**
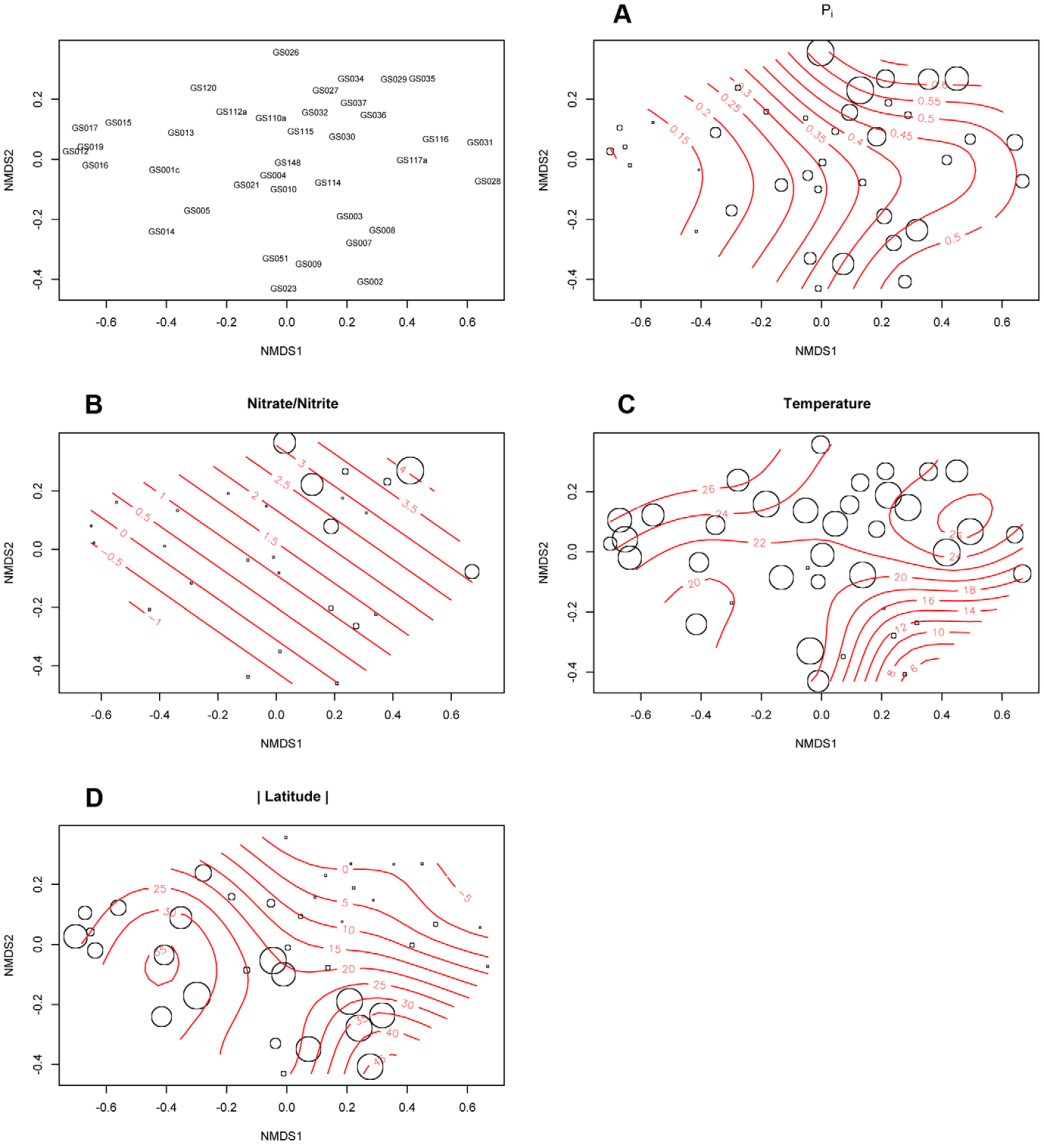
Non-metric Multidimensional Scaling plot of GOS sites phosphate metabolism gene abundance normalized to effective sequence counts. 2D Stress: 0.15. (A–D) represent bubble plots of environmental variables for (A) estimated P_i_ concentration from World Ocean Database (WOD); (B) estimated Nitrate/Nitrite concentration from WOD; (C) Surface temperature measured at time of sampling; (D) Absolute latitude of sample sites (i.e. distance from equator). Red contour lines represent a smooth fitted surface of estimated P_i_ concentrations from the World Ocean Database for each site, fitted using a generalized additive model using the R function ordisurf.

**Figure 6:**
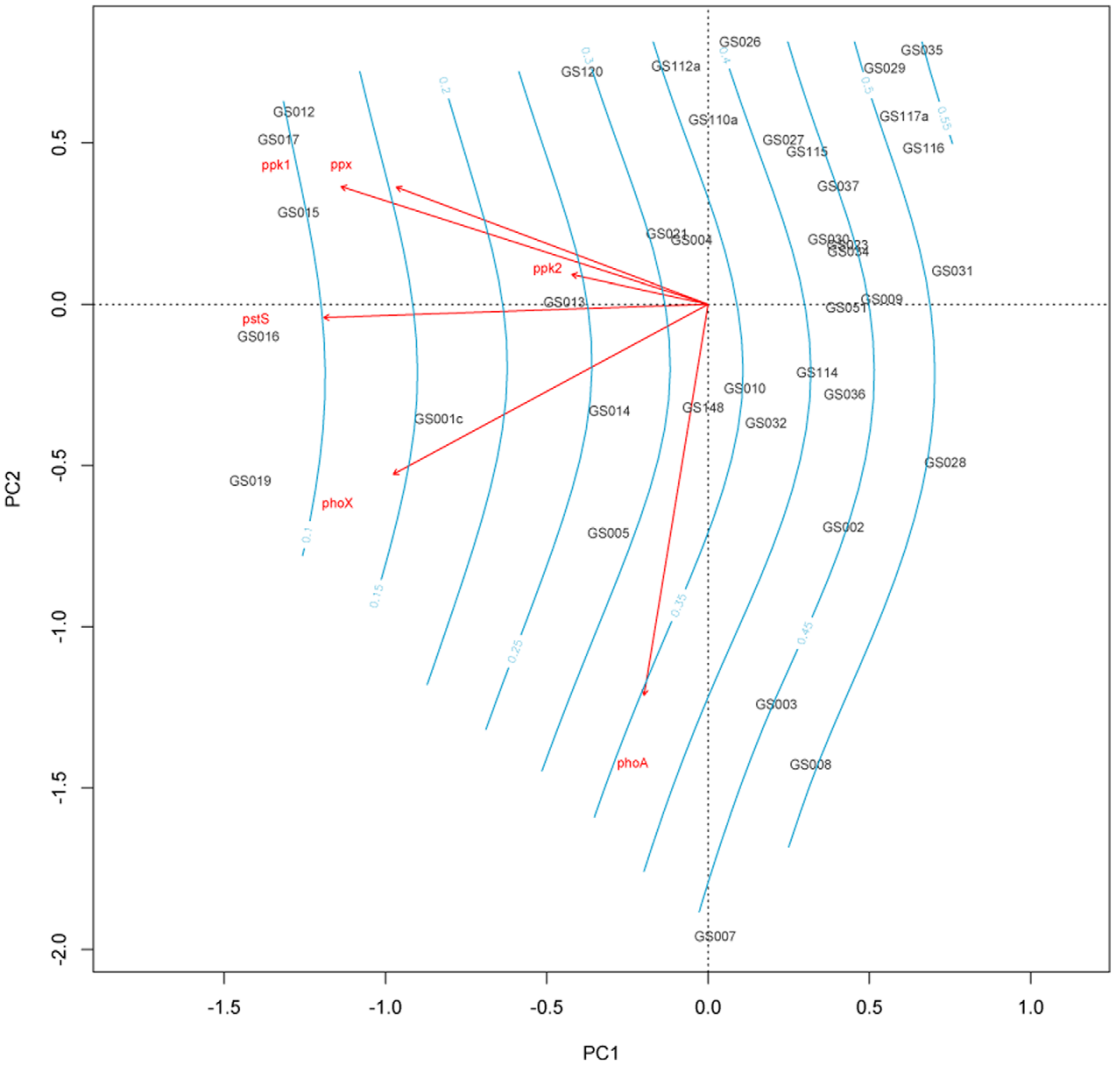
Biplot of principal component analysis of phosphate metabolism gene abundance normalized to effective sequence counts in GOS sites, overlaid with a smooth fitted surface of estimated P_i_ concentrations from the World Ocean Database for each site (blue), fitted using a generalized additive model using the R function ordisurf. Vector inertia is equal to correlation and scaled with optimum relation to sites. Gene sampling frequency was scaled to unit variance. PC1 explained 45% of the total correlation.

To investigate how abundances of polyphosphate genes, alkaline phosphatases and *pstS* changed between GOS samples, the total number of isofunctional homologs identified by IHAT for each gene at each site was normalized by effective sequence counts. A Bray-Curtis distance-matrix was then created and plotted as a Non-metric multidimensional scaling (NMDS) plot using the metaMDS function in the R vegan package [51]. Initial analysis included the hypersaline site, GS033, the freshwater site, GS020 and GS000a from the pilot study. **Figure S1** shows that these sites had very different profiles compared to other marine sites, forming a distinct cluster in a dendrogram of group-average hierarchical clustering from a Bray-Curtis distance matrix of phosphate gene abundance normalized to effective sequence counts. SIMPROF testing of this dendrogram in PRIMER v6 [52] revealed that GS000a and GS033 were not significantly different (91.7% similarity, 5% significance level, 999 permutations). Consequently, they were removed from subsequent analysis. NMDS plots of the remaining sites overlaid with environmental variables revealed a correlation between P_i_ concentrations estimated from the World Oceans Database (WOD) and ordination of sites along the primary axis (**Figure 5**). Environmental variables were fitted as smooth surfaces created using a generalized additive model via the ordisurf function in the R vegan package. Surfaces with a gradient parallel and equally spaced to the vector of the environmental variable represent a linear relationship between ordination and the environmental variable (represented by a bubbleplot). **Figure 5** also shows a linear response in ordination for estimated nitrate/nitrite concentrations, although 11 sites were removed from the analysis due to a lack of measurements in the WOD (GS012, GS017, GS031, GS032, GS051, GS114, GS115, GS116, GS117a, GS120, GS148). No such linear response was found for temperature or absolute latitude. Principal Components Analysis was performed using the rda function in the R vegan package. Gene sampling frequencies were scaled by unit variance to avoid domination by genes with high abundance and high variance. The PCA plot was overlaid with a fitted smooth surface of estimated P_i_ concentrations (**Figure 6**). Inertia was equated to correlation, with the primary principal component (PC1) axis explaining 45% of the total correlation and the secondary principal axis (PC2) explaining 19% of the total correlation. Unsurprisingly, there was a near linear response between *pstS* sampling frequency and estimated P_i_ concentration. Site ordination across PC1 was largely driven by *pstS*, *ppk1*, *ppx* and to a lesser extent, *phoX*. *phoA* and *ppk2* had little effect on ordination across PC1. Interestingly, *phoA* sampling frequency had a large effect on ordination across PC2, which showed a linear relationship with temperature estimated from WOD and latitude in environmental variable surface plots (data not shown).

For *ppk1*, *ppk2* and *ppx*, species composition of isofunctional homologs identified as reciprocal best-hit in STRING across the GOS samples was analyzed using SIMPROF. Between sites there was a paucity of shared species, known to affect Bray-Curtis distance measures [53]. Therefore species abundances between sites were compared using Manhattan distance over log(X+1) transformed data. SIMPROF analysis of *ppk1* species composition showed GS012, GS019, GS015, GS032 and GS013 were single-member clusters, while GS016 and GS017 also formed a separate cluster (5% significance level, 999 permutations) For *ppx*, GS015 and GS019 formed a cluster as did GS017. All other samples were clustered together. *ppk2* formed two significantly different clusters, with GS001c separated from all other samples, which were clustered together (**Figure S2**). *ppk1* isofunctional homologs were identified from a wide range of species in STRING, with the five most abundant species (*Gramella forsetii* KT0803, *Prochlorococcus marinus* MIT9312, *Flavobacterium johnsoniae* ATCC17061, *Prochlorococcus marinus* AS9601 and *Mesorhizobium sp.* BNC1) representing 171 of 485 *ppk1* homologs (∼35%) across all GOS samples. *ppx* was also broadly represented, with the five most abundant species (*Prochlorococcus marinus* MIT9301, *Clostridium difficile* 630, *Flav. psychrophilum* ATCC 49511, *Prochlorococcus marinus* AS9601, *Rugeria sp.* TM1040) representing 158 of 534 (29.6%) homologs. Conversely, *ppk2* homologs were dominated by two species in STRING: *Sulfurovum sp*. NBC37-1 (166 hits, 33.9%) and *Ruegeria pomeroyi* (55 hits, 11.8%). The lack of polyphosphate gene homologs from the ubiquitous *Can.* “P. ubique” HTCC7211 in the GOS samples is due the absence of its genome in version 8.2 of the STRING database.

## Discussion

Evaluation of IHAT in comparison to analysis using TBLASTN and HMMER3 against an artificial dataset of randomly sampled fragments from *Can.* “P. ubique” HTCC7211 revealed that overall, IHAT annotation is highly accurate, successfully identifying all fragments sampled from a particular gene, with no ‘false positives’ from other genes in the genome which may share ancestral homologous conserved domains. The use of *Can.* “P. ubique” HTCC7211 as a source for an artificial dataset had several advantages. Firstly, SAR11 genomes are highly streamlined as a result of nutrient-limited selective pressure, with few multi-copy genes [54]. Therefore, genomic fragments assigned to a particular gene by IHAT are unlikely to come from an uncharacterized isofunctional homolog, outside the loci of the known gene. The small genome size of *Can.* “P. ubique” HTCC7211 (∼1.46 Mbp) also allows high coverage of the genome with a relatively small number of fragments, reducing computational costs. The low guanine-cytosine (GC) content of SAR11 (29% in *Can.* “P. ubique” HTCC7211) results in skewed nucleotide coding sequences for genes (e.g. *Can.* “P. ubique” HTCC7211 *pstS* has a best-hit in nr to *pstS* of *Can.* “P. ubique” HTCC1062 with an expect-score of 1×10^−102^, dropping to 2×10^−24^ for the second best hit to *pstS* in *Arcobacter butzleri* RM4018). SAR11 genes therefore represent an extreme of nucleotide coding for a given isofunctional homolog and consequently the best test for the effectiveness of a new annotation tool. Finally, the genome of *Can.* “P. ubique” HTCC7211 was not included in STRING v 8.2, so any bias resulting from exact matches to a particular gene in the database were avoided. The results of this evaluation, and subsequent analysis of the GOS samples showed that the four criteria for isofunctional homolog annotation were met by IHAT.

The retrieval of all fragments for each gene with no false positives from the artificial dataset, coupled with the lack of correlation between 33 of 35 single copy marker gene abundance, and estimated phosphate concentration in GOS samples suggests that IHAT worked equally well on highly conserved and less conserved genes. The significant correlation of *hisS* and *leuS* with phosphate concentration was surprising as normalization of data using effective sequence counts, derived from effective genome size, which is in turn based off single-copy marker gene abundance, should effectively remove any correlation between single copy marker gene abundance and environmental variables. However, their *p*-values were only just below a significance cutoff of 0.05 (0.02 and 0.03 respectively). Conversely, the *recA* generalized linear model showed little difference to the null model (*p*-value  = 0.93), whereas that of *pstS* showed a highly significant difference (*p*-value  = 3.08×10^−6^) from the null model (**Figure 4**). Therefore, the significant correlation of *hisS* and *leuS* may have been a result of random chance.

Identification of homologs by IHAT was equally successful across both 1000 bp and 350 bp artificial datasets, suggesting that it is unaffected by reduced sequence size and thus suitable for analysis of both Sanger and 454 GS-FLX pyrosequenced reads. Evaluation of IHAT also revealed two advantages over HMMER3 for calculating the abundance of isofunctional homologs in metagenomic datasets. HMMER3 was highly sensitive to distant homology between conserved domains and thus returned many statistically significant homologs when searching for genes involved in nutrient transport, which tended to contain a large number of ATP binding domains, P-loops and other ubiquitous domains. Indeed, it must be stressed that this is the strength of HMMER3 and the retrieval of significant matches from other genes in the artificial dataset should be classed as a failure of its application as an annotation tool without a reciprocal check, rather than a weakness of the software. However, one advantage of IHAT is that it can be run against nucleotide sequences whereas HMMER3 is currently limited to amino acid sequences [33]. Translating nucleotide sequences into their amino acid counterparts using tools such as orf_finder prior to annotation suffers from insensitivity to frameshift mutations introduced during sequencing, whereas analysis of the nucleotide sequence directly using tools such as PSI-BLAST [34] can account for this frameshift by introducing a penalized gap.

The use of matching COG numbers as a reciprocal check of isofunctional homology against a database of known, fully sequenced genomes appeared to successfully filter out hits to genes that happened to share a conserved domain. This method has a major advantage over reciprocal BLAST vs. a database such as the NCBI nr/nt databases: With the current exponential increase in sequence information from an ever-increasing range of environments [55], the number of sequences in the nr/nt databases associated with unknown/uncultured bacteria and their hypothetical proteins will increase dramatically. As a result, reciprocal best-hit tests will be less likely to return a meaningful result as their top hit and will require an arbitrary cutoff of how many results should be parsed before rejecting a putative homolog, proportional to the number of unidentified sequences in the database. In comparison, the number of COG families is believed to be relatively limited. While the original work of Tatusov and colleagues somewhat underestimated the upper limit of families (1000 families vs. the >5000 families currently in the COG database) [40], the number of families will remain vastly smaller than the total number of non-redundant sequences. Therefore, even as the number of sequenced genomes in STRING increases, matching of COG family against the top hit of a reciprocal BLAST should remain consistently stringent.

Analysis of the GOS datasets for isofunctional homologs of polyphosphate metabolism genes revealed that *ppk1* and *ppx* in particular may contribute to the survival of phosphate-limitation in bacterial communities associated with oligotrophic environments. This finding supports recent research by Orchard and colleagues [56], which demonstrated polyphosphate accumulation in the abundant marine cyanobacteria *Trichodesmium* in P_i_-limited environments. The importance of *pstS* in P_i_-limitation survival has been well documented in both *Prochlorococcus spp.* [57], [58] and SAR11 [24], with *Prochlorococcus spp.* increasing the copy number of *pstS* once available P_i_ concentrations drop below 0.1 µM [58]. This dependence can clearly be seen in **Figure 4**, with *pstS* abundance dropping rapidly once average P_i_ concentrations exceed 0.2 µM. *pstS* abundance also showed a clear negative correlation with the ordination of GOS sites along the primary principal component axis in **Figure 6**, with a near-linear correlation to overlaid P_i_ concentrations. Both *ppk1* and *ppx* also showed strong negative correlation along this axis and results of generalized linear models suggested a highly significant negative correlation between their abundance and available P_i_ concentration. Whether *ppk1* is used to store P_i_ as polyphosphate for surviving periods of extreme P_i_-limitation, or whether it is used to drive transport equilibria by acting as a phosphate sink to maximize uptake requires further elucidation through laboratory work. However, this research indicates that the ability to synthesize polyphosphate and then subsequently degrade it to P_i_ via Ppx and/or via the reverse reaction of Ppk1 is not only retained in genomically streamlined strains, such as all 12 currently sequenced *Prochlorococcus spp.* genomes and *Can.* “P. ubique” HTCC7211 [54], [59], but is also found across a range of other species. Interestingly, the abundance of *ppk2* was not significantly correlated to average P_i_ concentrations, nor did it have a significant impact on ordination of GOS sites in NMDS. Unlike *ppk1* and *ppx*, abundance of *ppk2* was dominated by two species, an Epsilonproteobacterium, *Sulfurovum sp*. NBC37-1, associated with hydrothermal vents [60] and *Ruegeria pomeroyi* DSS-3, a pelagic member of the *Roseobacter* clade [61]. The lack of correlation between *ppk2* abundance and any of the measured or estimated environmental variables suggested that the factors driving the maintenance of *ppk2* in the genomes of marine bacteria have not been identified in this study. The annotation of *ppk2* to *Sulfurovum sp.* NBC37-1 and *ppx* to *Clostridium difficile* 630 from marine surface water samples highlights the fact that speciation of isofunctional homologs based on reciprocal best-hit analysis is limited to the number of species currently available in the STRING database. Such taxonomic assignations should therefore be broadly interpreted alongside known community composition from 16S rRNA studies. The importance of both *ppx* and *ppk2* to *Ruegeria spp.* (5^th^ most abundant *ppx* homolog and 2^nd^ most abundant *ppk2* homolog) is unsurprising, as members of this genus have been classified as ubiquitous ‘opportunitrophs’, with significantly elevated numbers of signal transduction genes and transport/binding proteins [61], to maximize nutrient uptake when they become available. The genome of *R. pomeroyi* lacks a homolog of *ppk1* but has three homologs of *ppk2*. One of these, protein SPO0224, was recently shown to favor the synthesis of polyphosphate from ATP rather than the catalysis of GDP phosphorylation using polyphosphate typically associated with *ppk2* [62]. It may be that for *R. pomeroyi*, this represents a specific adaptation, perhaps via post-translational functional modification of SPO0224, to utilize polyphosphate for maximal nutrient uptake. Further work is required to elucidate the role of this unusual *ppk2* homolog in *R. pomeroyi*.

The relative importance of the alkaline phosphatase encoded by *phoX* compared to *phoA* to ordination of GOS sites in an NMDS, and the significant correlation between *phoX* abundance and P_i_ not seen for *phoA*, confirms the findings of previous studies that indicated *phoX* was more abundant in marine metagenomic samples than *phoA*. PhoX is activated by the binding of Ca^2+^, in contrast to PhoA, which relies on Zn^2+^, typically available at subnanomolar concentrations in marine environments. [4]. *phoA* abundance was strongly correlated with the secondary principal component axis in **Figure 6**. This axis showed a non-linear relationship with latitude, suggesting that *phoA* may be more abundant at higher latitudes. Regulation of *phoA* is traditionally assumed to be tightly controlled by P_i_ availability, but the lack of correlation with P_i_ in this study may suggest other modes of regulation in marine systems.

In conclusion, IHAT is a novel pipeline for the annotation of isofunctional homologs that meets the specified criteria of accuracy. It enables estimation of the relative abundance of single and multi-domain homologs, free from expect-value cutoff bias, in both Sanger and 454 GS-FLX pyrosequenced metagenomic datasets. Reciprocal checking of the COG assignment of the best-hit from a BLASTX query against the STRING database provides not only an accurate way of filtering out heterofunctional homologs which share an ancestral conserved domain, but also yields broad taxonomic identification of the homolog for analysis of community composition. IHAT analysis of the GOS dataset has shown that polyphosphate metabolism via the Ppk1 and Ppx proteins may contribute to the survival of oligotrophs under conditions of low P_i_ availability. This research encourages further study of this largely overlooked polymer in marine bacterioplankton.

## Materials and Methods

### Investigation of the effect of expect-value cutoff for different genes

To investigate how expect-value cutoff with TBLASTN affected the number of identified homologs for different genes, a nucleotide database was created by combining all GOS datasets into a single FASTA file and then randomly subsampling to 46,052 sequences using the daisychopper (http://www.genomics.ceh.ac.uk/GeneSwytch/Tools.html) tool. A local alignment homology search was then performed against this database using amino acid sequences of *ppk1*, *ppk2*, *ppx*, *recA*, *gyrB* and *rpoB* as query sequences for TBLASTN at expect-value cutoffs log decremented from 10^−5^ to 10^−100^. Sequences that returned multiple High-scoring Sequence Pairs (HSPs) were counted as a single hit.

### Estimation of nutrient values for GOS Samples

To overcome the lack of nutrient metadata associated with GOS samples, average monthly or annual values for each sample site have previously been extrapolated from the World Ocean Database [6], [58], [63]. A similar method was used for this study. For each GOS sample site nutrient concentrations were downloaded from the World Ocean Database for a 1°×1° longitudinal and latitudinal square, centered on the original sample site. Measurements that were extreme outliers (e.g. phosphate concentrations >15 mM) were removed. Sites that had fewer than 3 records remaining were dropped from the analysis (**Table S1**). Average monthly values per site were not significantly different, so for each nutrient, the annual distribution was analyzed. Such distributions tended to be positively skewed so median rather than mean values were calculated.

### Preparation of the GOS samples for analysis

To overcome sampling bias between GOS datasets, poorly sequenced datasets were removed (e.g. GS113: 718 reads, GS114: 9741 reads) and each remaining dataset was randomly resampled to the size of the smallest remaining dataset (GS120 - 46,052 sequences) using the daisychopper (http://www.genomics.ceh.ac.uk/GeneSwytch/Tools.html) tool.

### Annotation of homologs using IHAT

IHAT is an application written in Python and is available for download under the Apache License Version 2.0 (http://www.apache.org/licenses/LICENSE-2.0) in a Subversion repository at http://code.google.com/p/homologfinder/. An overview of the application is shown in **Figure 2**. IHAT is run using two command line arguments: ‘–geneList’ points to a file containing the query genes and ‘–dbList’ points to a file containing the databases to be searched. Each gene/database combination is handled by a queue of threaded ‘worker’ objects to maximise throughput. To search for homologs for a particular gene, IHAT requires two pieces of information: (i) the Clustered Orthologous Group (COG) to which the protein encoded by the gene belongs and (ii) a hidden markov model (HMM) describing the primary structure of the protein. For each of the genes analyzed using IHAT, protein sequences were obtained from the National Center for Biotechnology Information (NCBI) website using accession numbers from publications where the encoded protein had been biochemically characterized, rather than using those based on predicted homology. The COG for this protein sequence was then identified using the STRING v 8.2 database (http://string.embl.de). Finally, the HMM for the protein was obtained using an HMM sequence search at the TIGRFAM web resource (http://www.jcvi.org/cms/research/projects/tigrfams/) and using the best-hit model (either a PFAM or a TIGRFAM model). The gene name and its associated COG and HMM name are stored in a file in tab separated format for parsing by the application. Once this information has been obtained, the homolog search proceeds via three main steps:

1. Generation of query information, including a whole-gene HMM, a consensus sequence from the STRING database and a checkpoint file.
2. PSI-BLAST search for homologs in the metagenome using products from (1).
3. Cross-check of homology against the STRING database using reciprocal best-hit analysis.

### Generation of query information

Due to the relatively short length of metagenomic fragments in comparison to full gene length, a fragment may span conserved domains that comprise a particular protein. For multi-domain proteins, use of a PFAM HMM, which typically models a single conserved domain of a protein, relies on sufficient coverage by the fragment of that domain to be recognized as homologous. Using an HMM for the full gene, rather than individual conserved domains, allows for greater specificity in annotation of homologs which span two conserved domains. Unlike PFAM HMMs, TIGRFAM HMMs consist of models for full-length proteins [64]. However, because the best-hit model for a protein sequence from TIGRFAM could either be a PFAM or a TIGRFAM model, full-length protein HMMs were constructed for each gene using protein sequences associated with a particular COG in the STRING database so as to avoid bias of using HMMs from different sources. For successful homolog calling using HMMs it is imperative that the input sequences used to construct the HMM are free from contamination by erroneously annotated sequences. Therefore, prior to construction of the HMM from STRING proteins, sequences assigned to a particular COG in STRING were subjected to two checks. Firstly, any sequences associated with multiple COGs (products of gene fusion etc.) were rejected. Remaining sequences were verified using hmmsearch from HMMER3 [33] using the HMM identified by TIGRFAM as the query model. Sequences outside of the inclusion threshold were rejected, ensuring that the sequences to be included in the creation of the STRING HMM matched either the full protein model (TIGRFAM HMM) or contained a ‘signature’ conserved domain for the protein (PFAM HMM). Filtering during this step is not limited to a specific HMM, allowing use of custom HMMs or other HMM datasets (e.g. FIGfams [65]) if required. Sequences that passed both checks where then aligned using MAFFT v.6 [66], a rapid, parallelized multiple sequence alignment program. The E-INS-I alignment protocol was used to improve alignment accuracy between distantly related homologs [67]. The alignment was then used to create an HMM using HMMER3 hmmbuild, which was in turn used to create a consensus sequence using hmmemit. The consensus sequence is then used to create a position-specific score matrix (PSSM) using blastpgp [34] with verified STRING COG sequences as a target database and the ‘-C’ option to output the PSSM as a checkfile, in a process similar to that used in the metaSHARK pipeline [68].

### Performing the PSI-BLAST

Using the checkfile generated above, metagenomic databases listed in the database file were then searched for homologs using PSI-TBLASTN, with no specified e-value cutoff with an effective database length set to 10^9^. Sequences of putative homologs identified by the PSI-TBLASTN were then extracted from the database in FASTA format using fastacmd (http://www.ncbi.nlm.nih.gov/blast) for use in the final stage.

### Cross-check of homology against the STRING database

**Figure 7:**
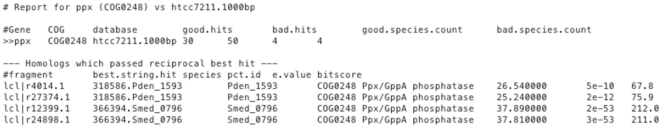
Example output from IHAT. Each output file contains a summary header followed by a list of successfully annotated homologs, followed by a list of sequences that failed the reciprocal BLAST against the STRING database.

Sequences of putative homologs retrieved by PSI-TBLASTN against the metagenomic database formed the query for a BLASTx (http://www.ncbi.nlm.nih.gov/blast) search against the full STRING v8.2 protein database. The best-hit for each sequence was then parsed from the output file and the associated COG and species of the best hit was recorded. If the associated COG of the best-hit from the STRING database matched the COG of the original gene, the metagenomic sequence was considered a successfully identified homolog. If the COG did not match, the sequence was recorded as a homolog that failed the reciprocal check. Results of the reciprocal BLAST analysis were then outputted to a report file (**Text S1**). For rapid parsing of reports, each file is headed with a summary of results (**Figure 7**).

### Testing the performance of IHAT

The sensitivity of IHAT to identify homologs was compared to traditional TBLASTN alignment analysis using expect-value cutoffs, and probabilistic inference analysis using HMMER3. In order to test the efficiency of homolog identification, a genomic dataset was required in which the absolute number of fragments from a particular gene was known. The MetaSim software [41] was used to create two such datasets to test IHAT over fragments 1000 bp (typical Sanger sequencing length) and 350 bp (typical 454 GSFLX pyrosequencing length) in length respectively. Fragments were sampled from the genome sequence of *Can.* “P. ubique” HTCC7211. Given the error rates used by MetaSim to simulate 454 (>3%) and Sanger (1∼2%) are much higher than reported error rates [45], the exact sampling model with default parameters was used. The databases consisted of 29,138 and 83,251 sequences respectively to give an approximate 20-fold coverage of the genome. The output from MetaSim was then parsed to find the locus from which each fragment was sampled and this was compared to the gene locus for each gene on the *Can.* “P. ubique” HTCC7211 genome. The total number of fragments for each gene was then calculated. A fragment was counted as sampled from a gene if it fell between the start and the end locus of the gene. This method avoided bias caused by differential conservation between genes at 5′ and 3′ ends. Comparison of methods was carried out across 11 genes encoding for products ranging from multi-domain proteins with regions of poor conservation to single-copy, highly conserved housekeeping proteins (**Table 1**). Two genes with no known homology in the *Can.* “P. ubique” HTCC7211 genome were also included to test for false positives. *ppk2* encodes a protein that catalyzes the phosphorylation of GDP to GTP using polyphosphate [69]. *creC* encodes for a sensor kinase that is believed to cross-regulate the phosphate limitation response in conjunction with PhoR [70]. For TBLASTN alignment analysis, amino acid sequences from *Pseudomonas aeruginosa* PAO1 for each of the 11 genes were used as a query in a TBLASTN (BLAST+ v2.2.23) search of the *Can.* “P. ubique” HTCC7211 datasets at expect cutoffs of 10^−35^, 10^−25^, 10^−15^ and 10^−5^. Hits were then parsed so that each identified homologous sequence was only included once per gene (i.e. multiple High-scoring Segment Pairs were negated). For analysis using HMMER3, each *Can* “P. ubique” HTCC7211 dataset was parsed using the orf_finder tool [45] into amino acid sequences and then searched using hmmsearch, at default settings with the STRING-derived HMM created by IHAT as the query model. Hits to multiple reading frames from the same fragment were counted as a single hit.

### Calculation of GOS Effective Sequence Counts

To remove the bias of average genome size on the likelihood of sampling a gene from a given metagenomic community, homolog counts were rescaled using effective sequence counts, a composite measure of sequence number and average community genome size [44], as described by Beszteri and colleagues [47].

## Supporting Information

Figure S1 (A) Non-metric Multidimensional Scaling plot of a Bray-Curtis distance matrix of phosphate metabolism gene abundances from GOS sites, normalized to effective sequence counts, showing the distance between phosphate metabolism gene profiles of hypersaline (GS033), freshwater (GS020) and pilot study site GS000a, compared to marine sites from the main study. 2D Stress: 0.12. (B) Dendrogram of group average agglomerative hierarchical clustering of the Bray-curtis distance matrix from (A) showing that GS020, GS033 and GS000a formed a distinct cluster.
(TIF)

Figure S2 Dendrograms of cluster analysis of log(X+1) transformed species abundances for (A) *ppk1* and (B) *ppx* using a Manhattan distance measure. Red lines represent clusters in which members are not significantly different from each other, as determined by a SIMPROF test (5% significance level, 999 permutations).
(TIF)

Table S1 **Calculated mean and median phosphate concentrations for each GOS site from the World Ocean Database.**
(XLS)

Text S1 **Sample output file from the IHAT pipeline.**
(TXT)
