# Supplementary figures and images for: Novel Analysis of Oceanic Surface Water Metagenomes Suggests Importance of Polyphosphate Metabolism in Oligotrophic Environments
Source: PLoS One. 2011 Jan 28;6(1):e16499. doi: 10.1371/journal.pone.0016499 (PMC3030594; doi:10.1371/journal.pone.0016499)

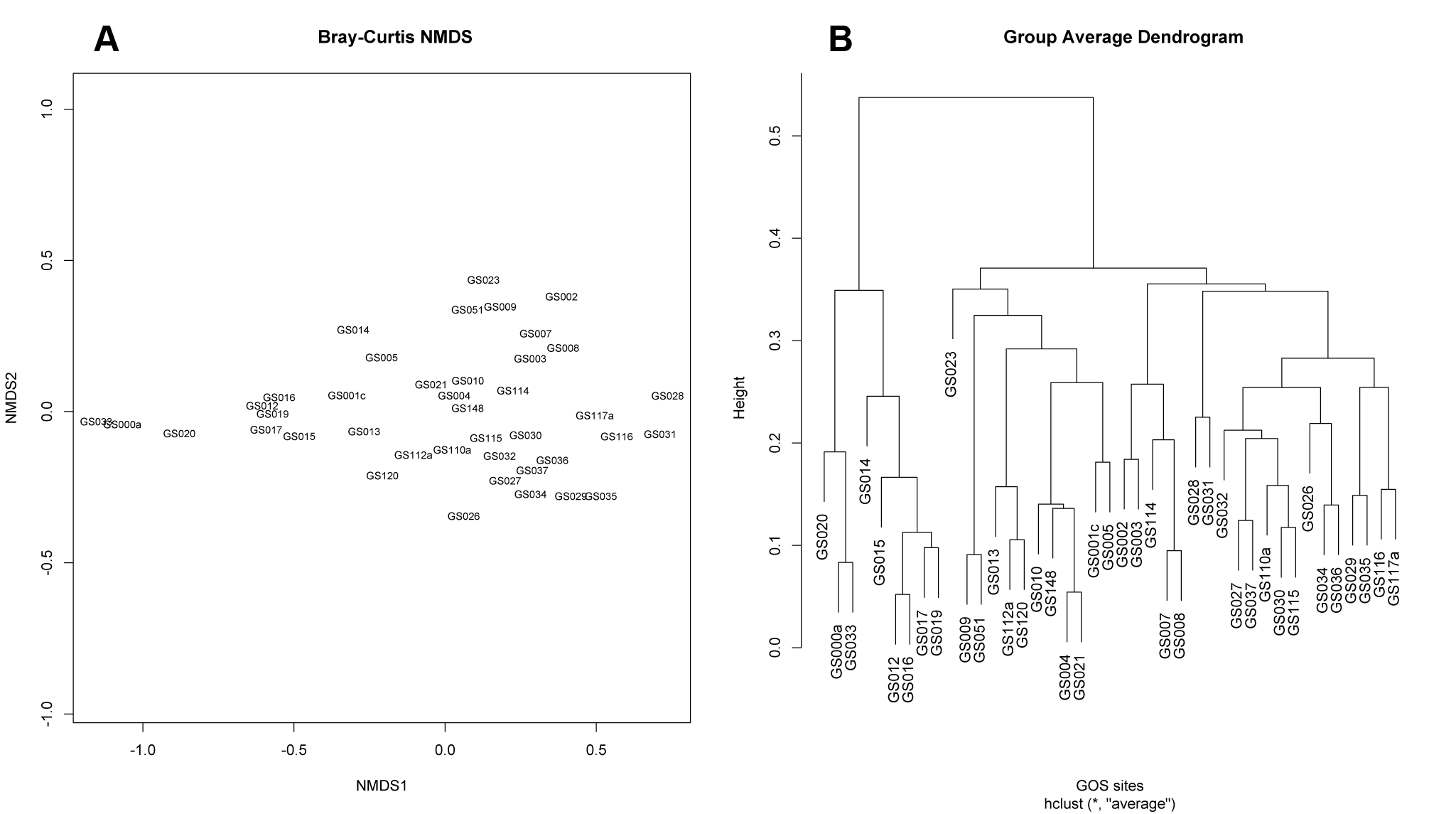

Supplement: Figure S1 — (A) Non-metric Multidimensional Scaling plot of a Bray-Curtis distance matrix of phosphate metabolism gene abundances from GOS sites, normalized to effective sequence counts, showing the distance between phosphate metabolism gene profiles of hypersaline (GS033), freshwater (GS020) and pilot study site GS000a, compared to marine sites from the main study. 2D Stress: 0.12. (B) Dendrogram of group average agglomerative hierarchical clustering of the Bray-curtis distance matrix from (A) showing that GS020, GS033 and GS000a formed a distinct cluster. (TIF) [file pone.0016499.s001.tif]

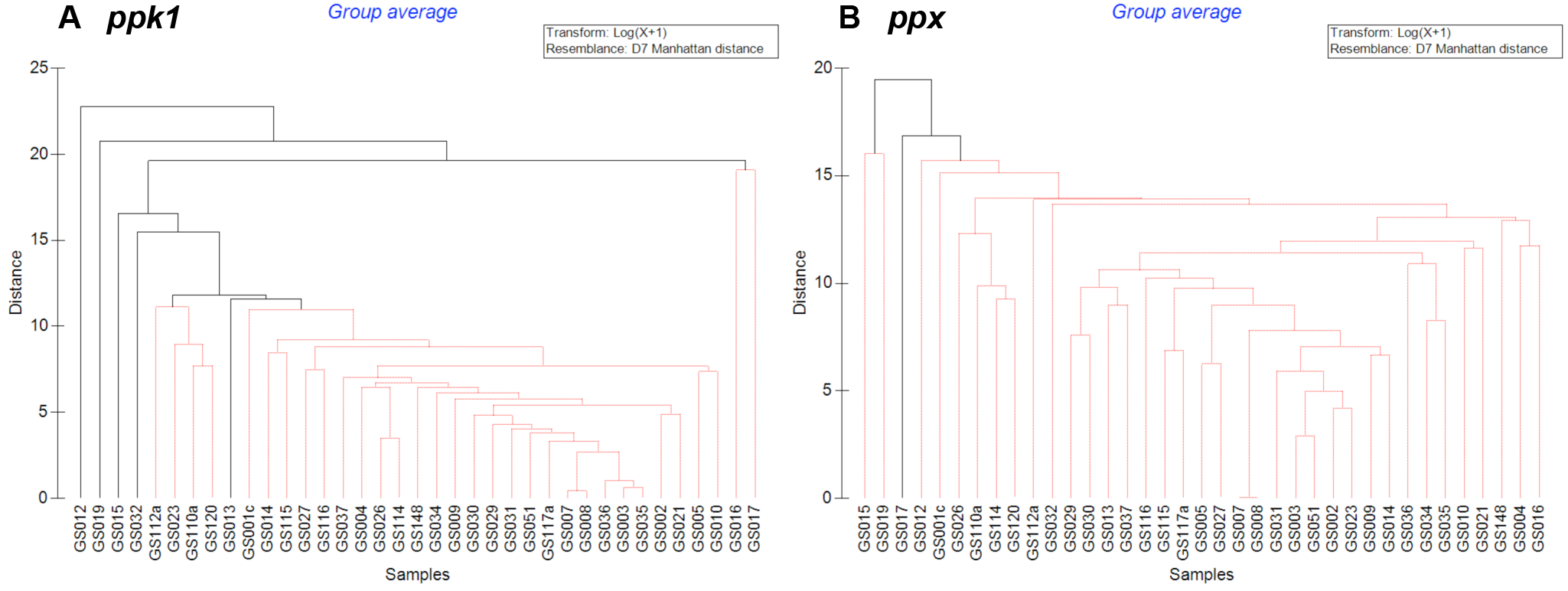

Supplement: Figure S2 — Dendrograms of cluster analysis of log(X+1) transformed species abundances for (A) ppk1 and (B) ppx using a Manhattan distance measure. Red lines represent clusters in which members are not significantly different from each other, as determined by a SIMPROF test (5% significance level, 999 permutations). (TIF) [file pone.0016499.s002.tif]
